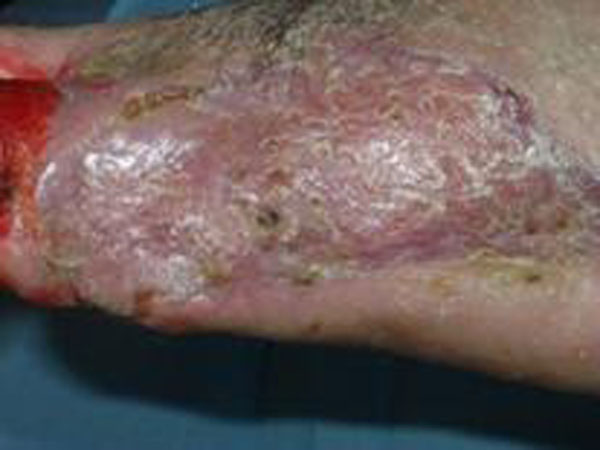# The use of negative pressure wound therapy (npwt) and dermal substitutes in the treatment of diabetic foot ulcers

**DOI:** 10.1186/1471-2318-11-S1-A39

**Published:** 2011-08-24

**Authors:** V Padovano Sorrentino, A Della Corte, A Fattopace, F Campitiello, M Ferretti, S Canonico

**Affiliations:** 1 Department Of Gerontology, Geriatrics And Metabolic Diseases, Second University Of Naples, Naples, Italy

## Background

The NPWT is becoming an important tool in the treatment of both acute and chronic wounds. The authors describe their initial experience using NPWT to fix a dermal substitute for preserving maximal foot length after surgical debridement in diabetic patients with foot lesions that were assessed for sensory-motor neuropathy and infection. The application of dressings to fix dermal templates can reduce shearing forces, restrict seroma and haematoma formation, simplify wound care and improve patient tolerance.

## Materials and methods

Two male patients, respectively 68 and 70 years old, with diabetes and peripheral neuropathy but without vascular dysfunctions were observed in our outpatient service in consequence of traumatic foot wounds with exposed bones and tendons. Antibiotic drugs were provided and NPWT was applied for three weeks after urgent surgical debridement. During this period, an increase of granulation tissue and decrease of nonviable tissue were observed. After three weeks in both patients minor amputations were performed because of bone necrosis. The patients continued NPWT (V1STA® Smith & Nephew) for 1 week until limited granulation of the wound bed was obtained. Subsequently an artificial graft with a dermal substitute (*INTEGRA***®***Dermal* Regeneration Template, SIAD) fixed by NPWT was performed. Later the wounds were closed with thin skin autografts.

## Results

In our initial experience, the use of NPWT to fix dermal templates reduced shearing forces and restricted seroma and haematoma formation. Moreover this practice achieved a faster granulating wound bed in order to prepare it for surgical closure techniques.

## Conclusions

The use of NPWT and dermal substitute make wound healing during the treatment of diabetic foot ulcers faster, decreases hospital stay and prevents major amputations. Moreover the application of NPWT dressings to fix dermal templates can simplify wound care and improve patient tolerance.

**Figure 1:**
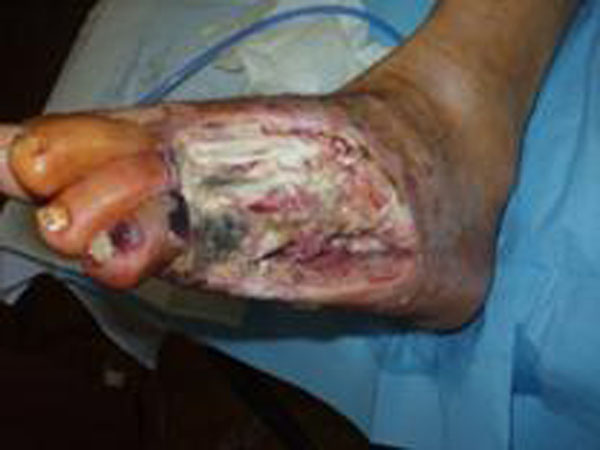


**Figure 2:**
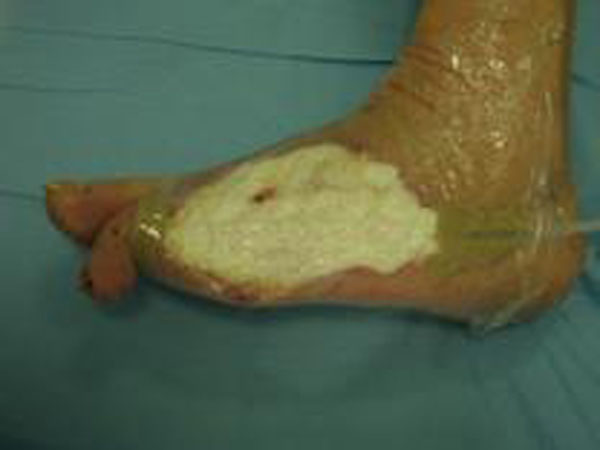


**Figure 3:**